# Machine Learning for Predicting Mortality Following Transcatheter Aortic Valve Replacement: A Scoping Review of Current Literature

**DOI:** 10.1016/j.cjco.2026.06.007

**Published:** 2026-07-23

**Authors:** Adham H. Elsherbini, Ibrahim Ahmed Cherif, Alisha Ahmed, Ali Haider, Ahmad Alaraki, Mohamed R. Gemae, Farzan Ansari, Ahmed A. Harby, Vipin Chaudhary, Yasir Abu-Omar, Mohammad El-Diasty

**Affiliations:** a Faculty of Medicine, University of Toronto, Toronto, Ontario, Canada; b Faculty of Health Sciences, Queen’s University, Kingston, Ontario, Canada; c Department of Opthalmology and Vision Sciences, University of Toronto, Toronto, Ontario, Canada; d Department of Emergency Medicine, McMaster University, Hamilton, Ontario, Canada; e The School of Computing, Queen’s University, Kingston, Ontario, Canada; f Computer and Data Sciences, Case Western Reserve University, Cleveland, Ohio, USA; g Harrington Heart and Vascular Institute, Cardiac Surgery Department, University Hospitals, Cleveland, Ohio, USA

**Keywords:** machine learning, mortality, transcatheter aortic valve replacement, cardiac surgery, artificial intelligence, postoperative complications, risk scores

## Abstract

This scoping review aims to consolidate the current evidence on the use of machine learning (ML) algorithms to predict mortality following transcatheter aortic valve replacement (TAVR) procedures. This review was conducted in accordance with Preferred Reporting Items for Systematic Reviews and Meta-Analysis (PRISMA) guidelines. Electronic databases, including Medline, Embase, Cochrane, and ClinicalTrials.gov, were searched for studies assessing the use of ML models for the prediction of mortality in patients undergoing TAVR. A total of 1076 citations were screened, of which 21 studies underwent full-text review. Ultimately, 11 studies comprising 101,030 patients were included for data extraction. Study sample sizes ranged from 178 to 54,739 patients, with reported mortality intervals ranging from 30 days to 5 years after surgery. The parameters that were included in these ML models included demographic and clinical variables, electrocardiographic findings, laboratory results, computed tomography scans, and the type and size of the selected TAVR device. A total of 13 ML models were assessed across the included studies. Random Forest appeared to be the highest performing ML model in predicting 30-day and 1-year survival following TAVR, with reported area under the receiver operating characteristic (AUROC) scores ranging from 0.66 to 0.97. In addition, the gradient-boosted machine seemed to outperform the CoreValve and TAVI2-SCORE in predicting 1-year survival. ML models may allow for accurate prediction of mortality following TAVR and may offer advantages over traditional clinical risk scores. However, limited datasets and variability in algorithm metric scores remain the main limitations to the implementation of these models in clinical practice.

## Résumé

Cette revue exploratoire vise à synthétiser les données actuelles concernant l’utilisation d’algorithmes d’apprentissage automatique (AA) pour prédire la mortalité à la suite d’une intervention de remplacement valvulaire aortique par cathéter (RVAC). Cette revue a été menée conformément aux lignes directrices PRISMA (*Preferred Reporting Items for Systematic Reviews and Meta-Analysis*). Des bases de données électroniques, notamment Medline, EMBASE, Cochrane et ClinicalTrials.gov, ont été consultées afin d’identifier les études évaluant l’utilisation de modèles d’AA pour la prédiction de la mortalité chez les patients bénéficiant d’un RVAC. Au total, 1 076 références ont été examinées, parmi lesquelles 21 études ont fait l’objet d’une analyse du texte intégral. Au final, 11 études portant sur 101 030 patients ont été retenues pour l’extraction des données. La taille des échantillons variait de 178 à 54 739 patients, avec des périodes de suivi de la mortalité allant de 30 jours à 5 ans après l’intervention. Les paramètres pris en compte dans ces modèles d’AA comprenaient des variables démographiques et cliniques, des données électrocardiographiques, des résultats de laboratoire, des tomodensitométries, ainsi que le type et la taille du dispositif de RVAC sélectionné. Au total, 13 modèles d’AA ont été évalués dans l’ensemble des études incluses. Le modèle « Random Forest » s’est avéré être le modèle d’AA le plus performant pour prédire la survie à 30 jours et à 1 an après un RVAC, avec des scores d’aire sous la courbe ROC compris entre 0,66 et 0,97. De plus, le modèle GBM (pour *gradient-boosted machine*) semblait surpasser les modèles CoreValve et TAVI2-SCORE dans la prédiction de la survie à 1 an. Les modèles d’AA pourraient permettre une prédiction précise de la mortalité après un RVAC et offrir des avantages par rapport aux scores de risque cliniques traditionnels. Cependant, le caractère limité des ensembles de données et la variabilité des scores de performance des algorithmes restent les principales contraintes à la mise en œuvre de ces modèles dans la pratique clinique.

Machine learning (ML), a subset of artificial intelligence (AI), equips machines with the capacity to learn from data, rather than through explicit programming for specific tasks. By analyzing and learning from data, machines improve their performance in various tasks, akin to gaining experience. Within the sphere of ML, we distinguish between supervised and unsupervised learning. Supervised learning utilizes mathematical models to discern patterns in labelled data, thereby guiding the prediction and mapping of new, unseen data. Clinically, this approach has been harnessed to predict patient outcomes across multiple cardiac surgery procedures,^1^, ^2^, ^3^ showcasing the profound impact of ML on healthcare and its potential for advancing medical prognostics. Transcatheter aortic valve replacement (TAVR) has become a widespread treatment option for patients with severe aortic valve stenosis.^4^, ^5^, ^6^ Since its approval by the US Food and Drug Administration (FDA) in 2011, a significant increase has occurred in patients undergoing TAVR procedures. Bana et al. estimated that 289,000 TAVR procedures will be performed annually by 2025, indicating a substantial increase from the 71,000 procedures that were performed in 2015.^7^

TAVR was initially indicated for the treatment of aortic stenosis in high surgical risk patients. However, more recent studies have shown its promising role in treatment of patients with intermediate and low surgical risk as well, which will likely result in an expansion of the pool of patients eligible for this technique.^8^ This expansion mandates the need for reliable risk assessment models that can be implemented to (i) accurately guide physicians during patient selection and procedural planning and (ii) help patients understand the expected outcomes of their planned procedure. In similar contexts, such as using ML to predict postoperative atrial fibrillation, advantageous results have been found due to the model’s ability to analyze complex correlation patterns between different variables.^9^

Currently, risk assessment of perioperative outcomes after TAVR is based mainly on clinical models that typically incorporate various demographic and clinical parameters in various logistics regression (LR) models.^10^ For instance, the **Euro**pean **S**ystem for **C**ardiac **O**perative **R**isk **E**valuation (EuroSCORE) II, which uses summed scores of weights derived from a an LR model, is a very popular risk assessment model initially developed for predicting perioperative risk in adult patients undergoing cardiac surgery.^11^ Other risk assessment scores, such as the Society of Thoracic Surgeons (STS) score and the Transcatheter Valve Therapy (TVT) score, also have been used; however, Al-Azizi et al. showed that the predictive ability of both scores is suboptimal.^12^ Due to the lack of a specific risk score, the EuroSCORE has been widely applied for the TAVR population.^13^ Similarly, some studies have developed algorithmic models that were used to predict the incidence of in-hospital stroke following TAVR. Nonetheless, these produced modest C statistic values, indicating that these models may not be sufficiently accurate for predicting this complication.^14^

It has been proposed that the application of supervised ML in the risk assessment of mortality after TAVR could potentially increase the accuracy of these models.^14^ Instead of considering set assumptions on data behaviour derived from historic TAVR cases, supervised ML models can be based on detecting and learning the underlying trends or patterns that govern the behaviour of these data.^15^ Also, given the predicted increase in the number of patients undergoing TAVR procedures, this will allow these potential models to be further trained, thus increasing their accuracy and improving their performance.^16^

Similar models already have been used to predict other adverse cardiovascular events such as coronary heart disease and atrial fibrillation from the progression of subclinical disease.^15^^,^^17^ These studies showed the effectiveness of ML models in predicting cardiovascular events from an initially asymptomatic population based on available predictors, offering the opportunity to identify a clear relationship between these predictors and specific cardiovascular events.^15^

The aim of this scoping review is to consolidate the current evidence on the role of different ML models in the prediction of mortality after TAVR procedures, with a particular focus on their applicability in routine clinical practice.

## Methodology

### Search strategy and study selection

This review was conducted in compliance with the Preferred Reporting Items for Systematic Reviews and Meta-Analysis—Extension for Scoping Reviews (PRISMA-ScR) guidelines.^16^ Requests for access to the extracted data or the data extraction template may be provided upon contact with the corresponding author. The following databases were electronically searched on December 2025 for primary studies evaluating the use of ML in predicting TAVR in patients undergoing cardiac surgical procedures: Medline, Embase, Cochrane, ClinicalTrials.gov, and Google Scholar. A combination of keywords—“machine learning,” “artificial intelligence,” “mortality,” and “transcatheter aortic valve replacement,” were used in the literature search. For consideration, papers had to be written in English, be primary studies including adults aged at least 18 years, and present results for an ML model used to predict mortality following TAVR.

### Study outcomes

Outcomes of interest included the reported specificity, sensitivity, and area under the receiver operating characteristic curve (AUROC) of the models that reported mortality after TAVR.

### Screening

Literature search results were uploaded to Covidence Systematic Review Software (Veritas Health Innovation, Melbourne, Australia. www.covidence.org).

### Data extraction

The author, publication date, study design, prevalence of mortality following TAVR, initial study population demographics, and pertinent results were among the data extracted. A table of study characteristics was created to extract and compile all the papers. Two independent blinded reviewers (A.H.E. and A.A.) assessed the quality of all of the studies using the Newcastle Ottawa Quality Assessment. All citations were screened primarily through the title and abstract stage by 2 independent blinded reviewers (A.H.E. and I.A.C.). A third reviewer (M.E.D.) resolved any conflicts. Studies that progressed past this stage underwent full-text screening by two reviewers (A.H.E. and I.A.C.). Studies that did not meet the inclusion criteria or lacked relevant outcome data were excluded.

## Results

### General results

A total of 1076 citations were screened. Of these, 21 studies underwent a full-text screening and 11 studies were ultimately included for their reporting of ML applications in predicting post-TAVR mortality (Fig. 1).^15^^,^^18^, ^19^, ^20^, ^21^, ^22^, ^23^, ^24^, ^25^, ^26^, ^27^ A total of 101,038 subjects were included in all the studies. The studies investigated transapical, transfemoral, and other alternative access approaches to TAVR. Each study used a different mortality assessment tool as well as ML models. All included studies reported on the predictive performance of their respective ML approaches. Study characteristics and study outcomes are summarized in Tables 1 and 2.

**Figure 1:**
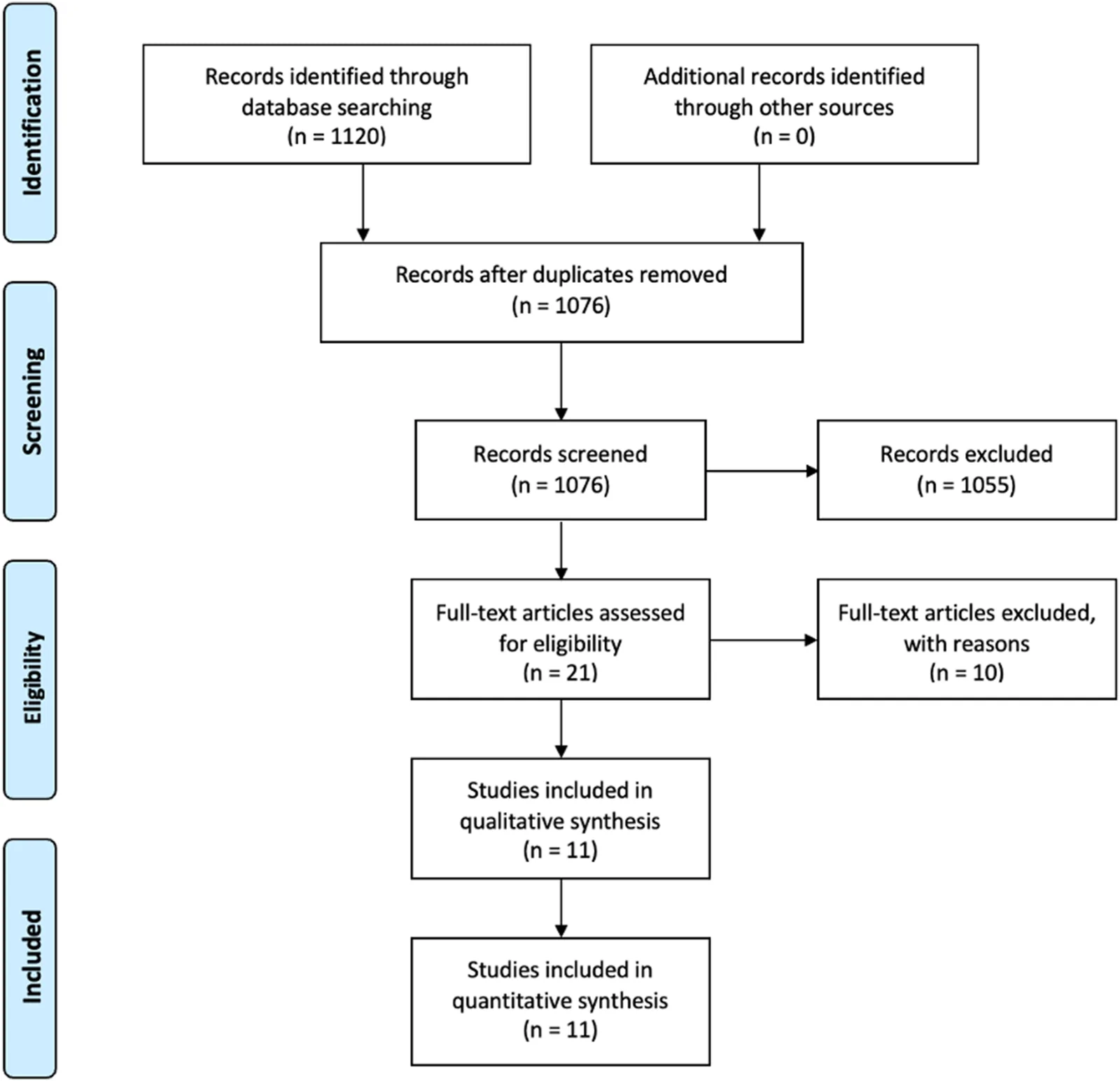
The workflow of isolation and culture in valvular interstitial cells. P4, passage 4; SAVR, surgical aortic valve replacement. Image created with BioRender.

**Table 1:** Study characteristics

Study	Country	Machine learning model(s)	Type of mortality and length (all cause, 30D, etc. (include all they report))	Type of TAVR (transapical, transfemoral, etc.)	Cross-validation	Diagnostic modality	Training sample size	Validation sample size	Test sample size
Alhwiti et al.^19^, (2023)	US	Dummy, LR, LightGBM, GBC, LDA, CatBoost	Fluid and electrolyte disorders, implantable cardioverter defibrillator, peripheral vascular disease, cardiac arrhythmias, chronic kidney disease, anemia, pulmonary circulation disorder, atrial fbrillation and flutter, chronic pulmonary disease, liver disease, and coagulopathy, cancer, carotid artery disease, dyslipidemia, valvular disease, smoking, coronary artery bypass graft, coronary artery disease, myocardial infarction, permanent pacemaker, percutaneous coronary intervention, and TIA	Transapical, endovascular	Stratified 5-fold cross-validation	Demographic variables, admission date, discharge date and status, primary and secondary International Classifcation of Diseases diagnoses, procedures, length of stay, etc	43,791	NA	10,948
Agasthi, et al.^18^ (2020)	US	Gradient boosting machine learning, CoreValve Model, TAVI2-SCORE Model	Cardiac power index, hemoglobin, systolic blood pressure, INR, diastolic blood pressure, body mass index, valve calcium score, serum creatinine, aortic annulus area, and albumin	NA	5-fold cross-validation repeated 10 times	Demographics, clinical, electrocardiography, computed tomography, and echocardiography data	1055	NA	NA
Kwiecinskiet al.^21^ (2023)	Poland	Gradient boosting machine learning	NA	TAVI (not specified)	(not specified)	Baseline characteristics of study participants. (clinical, history)	NA	823	604
Gomes et al.^20^ (2021)	Germany	ANN, RF, support vector machine, receiver operating characteristic for the STS score	Stroke/TIA, major vascular complications, PVL and new pacemaker implantations	Transfemoral	Nested 5-fold cross validation	Demographics and comorbidities, laboratory examinations, electrocardiogram findings, echocardiograms, and computed tomography scan reports, as well as the choice of valve type and size	452	NA	NA
Hernandez-Suarez et al.^15^ (2019)	US	LR, ANN, NB, RF, and SVM	Heart failure, diabetes mellitus, hypertension, obesity, peripheral vascular disease, chronic kidney disease, anemia, chronic pulmonary disease, coagulopathy, liver disease, cancer, prior TIA/stroke, atrial fibrilation, carotid artery disease, cardiogenic shock, cardiac arrest, major bleeding, vascular complications, AKI, sepsis	Transapical	NA	Prior history of heart failure, chronic kidney disease, coa- gulopathy, other valvular diseases, pulmonary circu- lation disorders, fluid/electrolyte disorders; be admitted to a hospital located in the Southern US; undergo TAVR via transapical approach; and have more hospital complications, such as stroke, myocardial infarction, conversion to SAVR, cardiogenic shock, cardiac arrest, major bleeding, vascular complications, acute kidney injury, and sepsis.	7615	3268	14,442
Lopes et al.^24^ (2019)	Netherlands	GTB, SVM, MLP, RFC, LR	Patient who died of a cardiovascular disease within 1 Y after the procedure	NA	Monte-Carlo cross-validation	Patient characteristics, medical history, symptoms, and test results prior to and after TAVI	70%	NA	30%
Mamprin, et al.^25^ (2021)	Netherlands	LR, RF, SVC, XGBoost, CatBoost, NN small, NN large, NN deep	1-Y mortality	Mainly transfemoral, some with other access sites	20-fold cross-validation	NA	NA	NA	NA
Mamprin et al.^26^ (2021)	Netherlands	GBDT, CatBoost CatBoost H., XGB, RFC, LR, SVM, GNB	1-Y mortality	Transfemoral with some other access sites	Stratified 10-fold cross-validation	NA	NA	NA	NA
Penso et al.^27^ (2021)	Italy	LR, RF, XGBoost, NN	5-Y mortality	Transfemoral, transapical	10-fold cross-validation	80 pre-TAVI variables	90%	NA	10%
Leha et. al.^22^ (2023)	Germany	TRIMpre, TRIMpost, aTRIMpost, aTRIMpre, STS	30-d mortality	Transfemoral	5-fold cross-validation	Patient record data (GARY, SwissTAVI)	22,283	6693	5864
Lertsanguansinchai et. al.^23^ (2023)	Thailand	Decision tree model	30-d and 1-Y mortality	Not specified (but it is TAVI)	10-fold cross-validation method	Baseline characteristics and preoperative clinical characteristics including symptoms of aortic stenosis, comorbidities, cardiovascular intervention history, medications, baseline 12-lead electrocardiography, baseline details of echocardiographic assessment before TAVI, the STS risk score, frailty score and mortality at 30-d and 1-Y from the electronic medical record	80% of variables with complete data	N/A	20%

All studies had a retrospective design.

AKI, acute kidney injury; ANN, artificial neural network; aTRIMpost, abridged Transcatheter Risk of Mortality Post-procedural score; aTRIMpre, abridged Transcatheter Risk of Mortality Pre-procedural score; CatBoost, categorical boosting; GBC, gradient boosting classifier; GARY, German Aortic Valve Registry; GBDT, gradient boosting decision tree; INR, international normailized ratio; LDA, linear discriminant analysis; LR, logistic regression; MLP, multilayer perceptron; NA, not applicable; NB, naïve Bayes; NN, neural network; PVL, paravalvular leak; RF, random forest; RFC, random forest classifier; SAVR, surgical aortic valve replacement; STS, Society of Thoracic Surgeons; SVM, support vector machine; TAVI, transcatheter aortic valve implantation; TAVR, transcatheter aortic valve replacement; TIA, transient ischemic attack; TRIMpost, Transcatheter Risk of Mortality Post-procedural score; TRIMpre, Transcatheter Risk of Mortality Pre-procedural score; XGB, extreme gradient boosting (XGBoost).

**Table 2:** Study findings

Study	Machine learning models	Specificity	Sensitivity	AUROC
Alhwiti et al.^19^ (2023)	Dummy, LR, LightGBM, GBC, LDA, CatBoost	Dummy (1), LR (0.771), LightGBM (0.677), GBC (0.810), LDA (0.785), CatBoost (0.795)	Dummy (0), LR (0.715), LightGBM (0.803), GBC (0.627), LDA (0.702), CatBoost (0.654)	Dummy (0.5), LR (0.817), LightGBM (0.825), GBG (0.824), LDA (0.816), Catboost (0.814)
Agasthi et al.^18^ (2020)	GBM Learning, CoreValve Model, TAVI2-SCORE Model	NA	NA	GBM Model (0.72), CoreValve model (0.53), TAVI2-SCORE model (0.55)
Kwiecinski, et al.^21^ (2023)	GBM Learning	NA	NA	NA
Gomes et al.^20^ (2021)	ANN, RF, SVM, Receiver Operating Characteristic for the STS Score	Specificity set to 0.9	ANN (0.8), RF (0.96), SVM (0.76), Receiver Operating Characteristic for the STS Score (0.08)	ANN (0.96), RF (0.97), SVM (0.94)
Hernandez-Suarez et al.^15^ (2019)	LR, ANN, NB, RF, and SVM	LR (0.839), ANN (0.042), NB (0.797), RF (0.167)	LR (0.877), ANN (0.944), NB (0.848), RF (0.963)	LR (0.92), ANN (0.85), NB (0.90), RF (0.90)
Lopes et al.^2^ (2019)	GTB, SVM, MLP, RFC, LR	NA	NA	GTB (0.69), SVM (0.69), MLP (0.66), RFC (0.70), LR (0.65)
Mamprin et al.^25^ (2021)	LR, RF, SVC, XGBoost, CatBoost, NN small, NN large, NN deep	NA	NA	(Internal validation, external validation): RF (0.67, 0.66), Catboost (0.65, 0.68), LR (0.65, 0.62)
Mamprin et al.^26^ (2021)	GBDT, CatBoost CatBoost H., XGB, RFC, LR, SVM, GNB	CatBoost (0.98), CatBoost H. (0.97), XGB (0.91), RFC (0.97), LR (0.72), SVM (0.74), GNB (0.20)	CatBoost (0.33), CatBoost H. (0.37), XGB (0.35), RFC (0.14), LR (0.53), SVM (0.49), GNB (0.87)	CatBoost (0.83), CatBoost H. (0.83), XGB (0.80), RFC (0.79), LR (0.60), SVM (0.58), GNB (0.57)
Penso et al.^27^ (2021)	LR, RF, XGBoost, Neural network	NA	MLP (0.71)	MLP (0.79)
Leha et. al.^22^ (2023)	TRIMpre, TRIMpost, aTRIMpost, aTRIMpre, STS	Internal Validation: TRIMpre (0.781), TRIMpost (0.725), aTRIMpost (0.788), aTRIMpre (0.0.733), STS (0.792)	Internal Validation: TRIMpre (0.587), TRIMpost (0.726), aTRIMpost (0.562), aTRIMpre (0.593), STS (0.514)	Internal Validation: TRIMpre = 0.74 aTRIMpre = 0.69 TRIMpost = 0.78 aTRIMpost = 0.74 STS = 0.69 External validation: TRIMpre = 0.69 aTRIMpre = 0.63 TRIMpost = 0.75 aTRIMpost = 0.67 STS = 0.67
Lertsanguansinchai et. al.^23^ (2023)	Decision tree model	NA	NA	0.71 (1-Y mortality), 0.83 (30-d mortality)

ANN, artificial neural network; aTRIMpost, abridged Transcatheter Risk of Mortality Post-procedural score; aTRIMpre, abridged Transcatheter Risk of Mortality Pre-procedural score; AUROC, area under the receiver operating characteristic curve; GBC, gradient boosting classifier; GBG, gradient boosting; GBM, gradient boosting machine; GBDT, gradient-boosting decision tree; GNB, Gaussian naïve Bayes; LDA, linear discriminant analysis; LightGBM, Light Gradient Boosting Machine; LR, logistic regression; MLP, multi-layer perceptron; NA, not applicable; NB, naive Bayes; RF, random forest; RFC, random forest classifier; STS, Society of Thoracic Surgeons; SVM, support vector machine; TRIMpost, Transcatheter Risk of Mortality Post-procedural score; TRIMpre, Transcatheter Risk of Mortality Pre-procedural score; XGB, extreme gradient boosting (XGBoost).

ML algorithms utilized in the studies included decision trees (DTs),^23^ LRs^15^^,^^19^^,^^24^^,^^25^^,^^27^ support vector machines (SVMs),^20^^,^^24^^,^^25^ multi-layer perceptron (MLP),^24^^,^^27^ gradient boosting DT (GBDT)^19^^,^^21^^,^^24^, ^25^, ^26^, ^27^ gradient boosted machine (GBM),^18^ K nearest neighbours (KNN),^19^ neural network (NN),^15^^,^^20^^,^^25^^,^^27^ random forest (RF) models^15^^,^^20^^,^^22^^,^^24^, ^25^, ^26^, ^27^ Naive Bayes (NB),^15^ Dummy classifier,^19^ linear discriminant models (LDAs),^19^ and gradient boosting classifier (GBC).^19^ Random oversampling was done in unbalanced training sets as necessary.^11^^,^^15^ These ML algorithms used a range of patient characteristics, such as age, sex, weight, body mass index, creatinine level, troponin T level, acute kidney injury, mean aortic pressure gradient, hematocrit, medication, and QRS duration, to predict the TAVR mortality.^15^^,^^18^, ^19^, ^20^, ^21^, ^22^, ^23^, ^24^, ^25^, ^26^, ^27^ Two studies included periprocedural variables such as the duration of the TAVR procedure, fluoroscopy time, total red blood cell concentrates (RBCs) transfused, and major and minor vascular complications as mortality predictors.^21^^,^^22^ Reported performance metrics, such as sensitivity and specificity, varied across the studies, ranging from 8% to 96% and 16.7% to 98%, respectively.^15^^,^^18^, ^19^, ^20^, ^21^, ^22^, ^23^, ^24^, ^25^, ^26^, ^27^ The area under the receiver operating characteristic curve (AUROC) values ranged from 53% to 97%, and the overall accuracy of the ML algorithms ranged from 27% to 97%.^15^^,^^18^, ^19^, ^20^, ^21^, ^22^, ^23^, ^24^, ^25^, ^26^, ^27^ Study outcomes are summarized in Supplemental Table S1.

### Model performance

Of the 13 ML models, RF models obtained the highest overall performance metrics for both short- and long-term post-TAVR mortality. For 30-day mortality, reported AUROCs ranged from 0.75 to 0.97, with sensitivity and specificity ranging from 0.726-0.963 and 0.167-0.725, respectively.^15^^,^^20^^,^^22^ For 1-year mortality, AUROC values ranged from 0.66 to 0.79, with reported sensitivity, specificity, and accuracy of 0.14, 0.97, and 0.88, respectively.^24^, ^25^, ^26^ Additionally, it was found that tree-based models were more accurate than other models in predicting mortality, outperforming LRs and MLP.^24^

One study used 2 RF ML models trained on pre-TAVR data (TRIMpre) and post-TAVR data (TRIMpost) to determine the pre-procedural and post-procedural TAVR risk of mortality, respectively.^22^ The TRIMpre model utilized 97 features and obtained an AUC of 0.74, whereas the TRIMpost used 155 features and obtained an AUC of 0.78.^22^ Additionally, XGBoost’s capacity to predict all-cause post-TAVR 1-year mortality is greater using both pre-procedural data and peri-procedural variables (AUC = 0.82), compared to utilizing only pre-procedural variables (AUC = 0.64).^21^

In their study, Alhwiti et al. found that AUC, balanced accuracy, sensitivity, precision, and F1 score improved as more features were used for prediction in all models.^19^ This was also the case for the RFmodels used by Leha et al.^22^ Moreover, with some models, notably LR and NB, the addition of features over a certain number yielded minimal performance improvements.^15^^,^^22^ Certain models, such as the ANN and RF models, resulted in a transient performance decline.^22^ Hernandez-Suarez et al. identified a transient decline in the AUC in certain models, such as the ANN and RF models, beyond the addition of 10 variables.^15^ However, the AUC of the RF model showed an upward trend beyond the addition of 20 variables, and beyond the addition of 30 variables in the ANN model.

## Discussion

In this review, we evaluated ML algorithms predicting post-TAVR mortality in adult patients. Based on the evaluated studies, ML models may have the potential to predict mortality with varying degrees of accuracy. The capabilities of ML algorithms can address some of the commonly observed shortcomings associated with the use of traditional risk models as TAVR becomes a more widely adopted procedure. TAVR has drastically changed the clinical care of aortic stenosis, with the TAVR procedures outnumbering surgical aortic valve replacement (SAVR) year-to-year in the US.^28^

Given the rising numbers of TAVR procedures, having an accurate and reliable risk stratification model of predicting mortality is important for both patient selection and reporting outcomes.^24^ Common mortality causes post-TAVR include infection/sepsis, heart failure, and multi-organ failure.^29^ ML models could direct postoperative care and follow-up for patients determined to be at risk for certain outcomes.^21^ The logistic EuroSCORE (LES) and Society of Thoracic Surgeons Predicted Risk of Mortality score are the risk scores most commonly used for the procedure, but both have limited TAVR-specificity, as they were initially intended for the SAVR population.^24^^,^^30^ Both algorithms also lack inclusion of factors that are often present in high-risk TAVR patients and are known to impact mortality, such as liver disease, previous chest radiation, neurocognitive dysfunction, and frailty. Although these factors may be common in the target patient population, a lack of common definitions and data on these factors when connecting them to TAVR has prohibited them from being included as covariates.^24^^,^^30^ The models evaluated by Alhwiti et al. took into account a history of liver disease as a baseline characteristic, a feature that was highlighted consistently by the models as important.^19^ The study by Lertsanguansinchai et al. included both frailty scores and liver disease when evaluating 30-day and 1-year post–transcatheter aortic valve implantation mortality.^23^

ML algorithms differ from traditional statistical models and can provide more accurate predictions of risk due to their ability to take into account more complex interactions and large amounts of data.^31^ Under the umbrella of ML, algorithms can be classified as having supervised, unsupervised, or reinforced learning. Many of the algorithms used for biomedical purposes are supervised learning algorithms, including KNN models, linear models, support vector machines, NB algorithms, DTs, RF models, GB models, and NNs.^31^ Commonly used starting points for biomedical research are logistic regression, supervised learning networks, artificial NNs, naive Bayes, random forest models, and support vector machine models.^15^^,^^31^

Developing a supervised learning model involves training an algorithm with labelled data—that is, input data with its corresponding outcome.^32^^,^^33^ Oftentimes, the training set is further split into *k* subsets for *k*-fold cross-validation. The model is trained on *k*–1 subsets, and evaluated on the remaining subset; this process is done *k* times, with the model being discarded each time, but the prediction errors are kept and eventually averaged to give a more accurate measure of the model’s predictive ability.^34^ From there, the algorithm is evaluated and validated using a testing dataset—the input data without the associated outcomes is given to the model, which can then iteratively improve until it is determined to have adequate accuracy. The testing set also serves to evaluate how well the trained model can be generalized to data outside the labelled set.^32^ In contrast, with an unsupervised model, including clustering algorithms, an algorithm’s objective is to identify connections and groupings without knowledge of the outcome. Within the healthcare context, supervised ML algorithms are typically more popular, particularly given their applicability for prediction.^33^ Of these supervised algorithms, LR and KNN models are relatively simple to use and implement. The NB and SVM models offer reliable findings, with the NB model being well-suited for larger data sets. The RF model is highly flexible, and automatically addresses missing values. XGBoost and LightGBM models, both DTB algorithms, can both be more challenging to comprehend. However, XGBoost can deal with missing values, and LightGBM can create more complicated trees and perform well with larger datasets **(**Supplemental Table S2**)**.

When predicting mortality in TAVR patients, an ML algorithm’s ability to integrate and discover hidden connections serves to address some of the drawbacks of traditional risk prediction models. Firstly, traditional risk scores, such as the Society of Thoracic Surgeons Predicted Risk of Mortality, were created for patients undergoing a surgical operation, whereas patients undergoing TAVR have a significantly different risk profile, likely with different postoperative outcomes. Traditional risk score models do not comprehensively represent all risk factors for TAVR, due to a lack of TAVR-specific relevant data when building these models.^30^ With an ML model, various diagnostic modalities can be taken into account, including electrocardiography, computed tomography, echocardiogram, clinical data, and demographic information, which can be integrated and through which patterns can be found to predict TAVR outcomes.^24^ Furthermore, ML algorithms are more equipped to deal with missing data. The included studies used numerous datasets and variables with which the ML models are trained, which are summarized in Table 1. Although the included variables are not uniform across models, all included some form of patient demographic information, and most studies included forms of clinical data, including electrocardiography and computed tomography findings. Both Alhwiti et al. and Hernandez-Suarez et al. highlight the limitation of using ICD codes to derive clinical information, as it may not represent possible risk factors as accurately as possible.^15^^,^^19^

In the study by Kwiecinski et al., an extreme gradient boosting model was developed, which had an AUROC of 0.82 for predicting all-cause mortality within a year after hospital discharge post-TAVR, outperforming the EuroSCORE II risk score’s AUROC of 0.57.^21^[21]. These findings highlight the ability of the algorithm to take into account many features, including the TAVR-specific comorbidities left out from traditional risk models built on data from SAVR patients.^21^

ML algorithms have been evaluated for a range of TAVR-related uses. In a study attempting to create an effective ML model to predict patient hospital charges, ML allowed the comparison of many variables and a breakdown of their associated impacts on overall cost, which could provide valuable data for healthcare providers and policymakers regarding the cost-effectiveness of this procedure.^35^ Multiple studies have reported ML models for predicting post-TAVR permanent pacemake (PPM) implantation with AUROCs ranging from 0.66-0.88, showing ML models have promise in this application.^36^ A study by Agasthi et al developed a RF gradient boosting ML model with an AUROC of 0.72 and high specificity, compared to the 0.54 AUROC of the PPM risk model.^37^ In addition, ML algorithms for predicting PPM implantation have been shown to provide similar potential benefits to algorithms predicting postoperative mortality for TAVR and a promising potential for effective risk stratification.^37^ Algorithms predicting mortality following TAVR could be improved by training them on larger, more complete datasets. Additionally, future models could include predictions for long-term outcomes, particularly beyond 1 year post-TAVR.

### Limitations of the study

The present study has some limitations. First, most of the included studies were retrospective in design. This design limits the ability to control for confounding variables and introduces potential biases related to data collection and patient selection. Second, the potential exists for overfitting in ML models in the included studies. Although these studies effectively utilize ML to predict various outcomes in patients undergoing TAVR, there is a risk that the models are overly tailored to the specific datasets used. This overfitting can limit the generalizability of the findings to broader populations, as evidenced in the diverse patient demographics and clinical settings of these studies. Third, considerable methodological discrepancies are present among the included studies, particularly in the variables and ML techniques employed. This heterogeneity limits the ability to perform direct comparisons between the findings of different studies. For instance, the variables deemed significant in one study might be absent or considered less critical in another, as seen in the differing predictive variables highlighted in each study. Finally, the studies predominantly focus on short-term outcomes, such as 1-year mortality post-TAVI. The lack of long-term data limits the understanding of the role of ML models in predicting long-term outcomes.

## Conclusion

Current evidence shows the promising role that ML can play in predicting mortality in patients undergoing TAVR procedures. When combined with clinical risk scores, ML may help improve preoperative risk stratification and patient management. More comprehensive studies are needed to better delineate the role of predictive models based on ML in routine clinical practice.

## Data Availability

All data are available upon request to the corresponding author.

## Ethics Statement

The research reported in this paper adhered to PRISMA-ScR guidelines.

## Patient Consent

The authors confirm that patient consent is not applicable to this article given that the study is a review.

## Funding Sources

The authors have no funding sources to declare.

## Disclosures

The authors have no conflicts of interest to disclose.

## Footnotes

To access the supplementary material accompanying this article, visit *CJC Open* at https://www.cjcopen.ca/ and at https://doi.org/10.1016/j.cjco.2026.06.007
