# Supplementary material for: Machine Learning for Predicting Mortality Following Transcatheter Aortic Valve Replacement: A Scoping Review of Current Literature
Source: CJC Open. 2026 Jul 23;8(10):1375–85. doi: 10.1016/j.cjco.2026.06.007 (PMC13654375; doi:10.1016/j.cjco.2026.06.007)
Supplement: Supplementary file 1 [file mmc1.pdf]

Supplemental Table S1. General Study Conclusions

Study	General Conclusion	significant risk factors	limitations
Theyab Alhwiti, Summer Aldrugh, Fadel M. Megahed. (2023)	Post-TAVR survival and death can be somewhat predicted using only administrative, preoperative data and several standard statistical/machine learning models without frequent retraining.	NA	(a) the lack of clinical data; (b) the surveillance bias phenomenon of "the more you look, the more you find," which can make the study of certain diagnoses/ complications invalid; and (c) the volume of data (i.e., big data) can make p values for statistical significance frequently much less than the typical cutoff of 0.058
Agasthi, Pradyumna et al.	machine learning was used to predict 1 year mortality among high/prohibitive surgical risk patients being selected for TAVR. Machine learning outperformed the TAVI2-SCORE and the CoreValve model in predicting survival 1 year post TAVR. CPI was found to be the best predictor of mortality out the 163 variables that were examined.	NA	(a) complexity of model and difficult interpreation impede reproducibility; (b) scoring system could be restricted by the availability of detailed information on underlying morbidities; (c) results were validated internally, not externally; (d) the study may not predict in-hospital mortality as it is designed to predict long term or 1-year mortality; (e) the model's application is limited in modest-risk and lower-risk patients, as the majority of the patients were at higher or prohibitive risk for SAVR;
Kwiecinski, Jacek et al.2023	Machine learning based on readily available clinical data allows accurate prediction of 1-year all-cause mortality following TAVI. The machine learning model could potentially be used to guide the intensity of patients' follow-up and post-discharge care after TAVI	NA	a) model used did not include scores that characterize patients' frailty, concomitant coronary artery disease, or the perception of their health status (b) risk for selection bias and residual confounding due to observational nature of study (c) study does not address need for pre-procedural tool for prediction of adverse outcomes
Gomes, Bruna et al.2020	Machine learning-derived prediction models seem to improve patient selection considering the outcome "intrahospital all-cause mortality". More research needs to be done to determine whether AI-derived risk models are more effective at predicting mortality compared to "one-size fits all" philosophies of the past	NA	(a) the low STS score and STS/ACC TAVR AUCs might be caused by its different internal validation cohort; (b) dataset was not particularly large
Hernandez-Suarez, Dagmar et al.2019	The good discrimination of this model reveals the potential of AI in the patient risk stratifi- cation process not just for TAVR but for any novel structural intervention. Further validation and application of ML into the day-to-day clinical practice is still warranted to better understand its true value in patients with severe aortic stenosis.	NA	(a) Inability to restrict variables pre- procedure versus post-procedure, which provides a dynamic nature to the NIS TAVR score; (b) the NIS is an observational cohort using ICD-9-CM and procedure codes, which relies on fidelity of hospital billing data, and deriving clinical informa- tion from ICD-9-CM codes is an inherent limitation; (c) because only patients undergoing TAVR between 2012 and 2015 were included in our study, our results may better represent the extreme and high surgical risk patient population; (d) ML model complexity and difficult interpretation may hinder its reproducibility and application into the standard practice.
Lopes et al.2019	RF was best at predicting mortality. Tree-based models were slightly more accurate than others in predicting mortality	NA	unbalanced measures (relatively small population that did not survive 1st year), many patients excluded because of missing daata
Mamprin, lopes, et al. 2021	NA	NA	NA
Mamprin, zelis, et al.2021	this approach shows promising predictive potential with respect to widespread prognostic risk scores	NA	NA

Penso et al.2021	ML approach for assessment of risk factors for long-term mortality after TAVI to improve clinical prognosis	NA	size of dataset was limited, single-centre study. Dataset included patients undergoing TAVI until 2014
Leha et. al.,2023	TRIM scores advance preprocedural and postprocedural risk assessment, integrating relevant information from the course of TAVI treatment with strong predictive performance	NA	a) study had to exclude cases due to missing endpoint annotations. (b) Features for models are incomplete as they rely on patient record data (c) There is a time gap between the analyzed cohort and future cases to be predicted
Lertsanguansinchai et. al.,2023	These machine learning models show excellent accuracy and have a better prediction for 30-day and 1-year mortality than the existing established TAVI predictive risk scores	Variables that predict 30-day mortality are a set of factors driven mainly by height, chronic lung disease, STS score, preoperative LVEF, age, and preoperative LVOT VTI. Variables that predict 1-year mortality are a set of factors consisting of preoperative LVEF, STS score, heart rate, systolic blood pressure, home oxygen use, serum creatinine level, and preoperative LVOT Vmax	a) At 30 days, a very small proportion of patients (7/178) died during this period and any existing model that inadvertently predict "all" survived could still gain very high accuracy (b) single-center study + models developed were validated internally (c) majority of patients in the study had high and intermediate risk (d) models developed from ML were complex and difficult to interpret

Supplemental Table S2. Benefits and Setbacks of ML models

ML Algorithm Model	Description of Model	Advantages	Limitations
Logistic regression (LR) **[38,39]**	• Uses flow properties, such as continuous, discrete, or hybrid, prior to linearly combining the inputs by passing them through a logistic function.<br>• Most widely used supervised ML algorithm as it produces more reliable results in large datasets.	• Associated with low variance.<br>• Offers probability for output.<br>• Simple to use, and training takes little time.	• May be modified to multiclass classification using a variety of applications but cannot naturally categorize multi-class data.<br>• Does not function well in the presence of interconnected attributes<br>• In situations where there are too few observations compared to features, logistic regression may result in overfitting.<br>• Regression models need relevant and biologically important independent predictor variables to be valid.<br>• Collinearity can cause errors or uncertainty in estimating effects.<br>• Variables must have consistent association magnitudes, and interactions between predictors must be considered for valid estimates.
K-nearest neighbor (KNN) **[40]**	• Simplest of the supervised ML algorithms<br>• To apply the algorithm, attribute vectors must be constructed.<br>• Next number of neighbourhoods is specified by the k parameter, which also determines the nearest neighbor that each data input should be assigned to.	• Simple to implement and comprehend since it is free of assumptions.<br>• Heuristic in nature.<br>• Swiftly adapts to input changes while being used in real-time.<br>• Could be easily utilized to solve multi-class classification problems.	• The pace of the algorithm slows down significantly as the amount of data increases.<br>• As the number of variables rises, it gets more challenging to obtain the desired output.<br>• It is impacted by outliers and is unable to handle missing numbers.<br>• The variable characteristics must all be stated in the same scale for the system to function effectively.
Naïve Bayes (NB) **[41,42]**	• Implements the conditional independence rule, which stipulates that all properties are independent variables and thus changes in each variable would do not impact others.<br>• Useful supervised ML algorithm when classifying larger data sets.	• Very reliable findings are produced using this relatively quick and adaptable model.<br>• Highly suited for larger data sets.<br>• Training doesn't require much of your time.<br>• Reduces unnecessary specifications to improve grading performance.	• To attain favourable results, large data records are required.<br>• Compares poorly to the other classifiers in terms of performance depending on the type of problem.<br>• Conditional Independence assumption may not always work due to feature dependence, causing issues like zero-probability and suboptimal binning with Multinomial Naive Bayes.<br>• Imbalanced data may not be handled well with Complement Naive Bayes.
Support vector machine (SVM) **[43,44]** 2026-06-21 8:22:00 PM2026-06-21 8:22:00 PM	• A supervised ML algorithm that is based on statistical learning theory.<br>• Used prominently for the classification of binary, multiclass or non-linear data.<br>• The foundation of SVM is the prediction of the decision function that can discriminate between classes.	• Produces reliable findings despite the lack of sufficient data.<br>• Particularly effective with unstructured data.<br>• Uses a handy kernel solution function to solve difficult problems.<br>• Is comparatively excellent at high dimensional data scaling.	• Choosing the right kernel solution function is often challenging.<br>• When working with extensive data sets, training takes a while.<br>• The model could be challenging to perceive and comprehend due to issues brought on by individual circumstances and varied weights.<br>• The contribution of each variable to the outcome varies because the variable weights are not constant.<br>• SVM struggles with complex classification problems.

Random forest (RF) **[45,46]**	• Simple supervised ML algorithm utilized for classification of data and generation of decision trees since it is not vulnerable to overfitting.<br>• When training this ML algorithm, it produces many decision trees from the subset of the problem and estimates each tree.<br>• The most voted estimations are used for classification.	• Because of the relationship between training and testing data, the likelihood of encountering a classifier that does not perform well is decreased.<br>• Extremely flexible with very high accuracy that is present even when a large proportion of the data are missing.<br>• Versatility in solving regression and classification problems.<br>• Handling both categorical and continuous variables<br>• Automatic addressing of missing values, no feature scaling requirement<br>• Efficient handling of non-linear parameters<br>• Robust to outliers and noise<br>• Stable in handling new data without significant impact on accuracy.	• Visually challenging to comprehend and interpret.<br>• Significantly more time-consuming and difficult to build than decision trees.<br>• Computation-intensive and the algorithm itself is less heuristic.
Extreme gradient boosting (XGBoost) **[47]**	• A scalable form of gradient boosting which combines outputs from trees to generate predictions.<br>• By building more trees, this supervised ML algorithm reduces the errors of the prior trees and as such the model increases in reliability as trees are added.	• When the data is clean, it can avoid overfitting.<br>• Can deal with missing values.<br>• Enables for cross-validation at each iteration of the process, maximizing the number of iterations.	• More challenging to comprehend compared to other linear algorithms.<br>• Data that is noisy could overfit.
Light gradient boosting machine (LightGBM) **[48,49]**	• Another type of supervised, decision tree-based ML algorithm that effectively implements aspects of the gradient boosting framework.<br>• Main difference is that it grows trees vertically instead of horizontally as other ML models would.	• Utilizes an optimization technique called histogram-based split finding, which accelerates the training process by dividing continuous feature values into discrete bins.<br>• Reduces memory use by switching continuous data to discrete bins.<br>• By using a leaf-wise tree growth strategy instead of a level-wise split approach (the primary element in getting greater accuracy), it creates far more complicated trees.<br>• With a considerable reduction in training time, it can perform just as well with larger datasets as other gradient boosting models	• Since it employs leaf-wise tree development to build deeper, more intricate trees, it can be more challenging to analyze and comprehend.<br>• Prone to overfitting, especially in small data sets
